# Utility of Multi-target Nested PCR and ELISPOT Assays for the Detection of Paucibacillary Leprosy: A Possible Conclusion of Clinical Laboratory Misdiagnosis

**DOI:** 10.3389/fcimb.2022.814413

**Published:** 2022-04-11

**Authors:** Haiqin Jiang, Ying Shi, Santosh Chokkakula, Wenyue Zhang, Siyu Long, Zhenzhen Wang, Wenming Kong, Heng Long, Limei Wu, Lihua Hu, Qiang Yao, Hongsheng Wang

**Affiliations:** ^1^ Department of Mycobacterium, Jiangsu Key Laboratory of Molecular Biology for Skin Diseases and STIs, Institute of Dermatology, Chinese Academy of Medical Sciences & Peking Union Medical College, Nanjing, China; ^2^ National Centre for STD and Leprosy Control, China CDC, Nanjing, China; ^3^ Centre for Global Health, School of Public Health, Nanjing Medical University, Nanjing, China; ^4^ Department of Microbiology, Chungbuk National University College of Medicine, and Medical Research Institute, Cheongju, South Korea; ^5^ Department of Leprosy Control, Zhejiang, Provincial Institute of Dermatology, Zhejiang, China; ^6^ Department of Leprosy Control, Wenshan institute of Dermatology, Wenshan, China

**Keywords:** diagnosis, PCR, nested PCR, ELISPOT, paucibacillary leprosy

## Abstract

The diagnosis of paucibacillary (PB) leprosy often possesses a diagnostic challenge, especially for pure neuritic and lesser skin lesions with the zero bacillary load, requiring a sensitive and accurate diagnostic tool. We have included 300 clinically diagnosed new leprosy cases (comprising 98 PB cases) and analyzed the sensitivity and specificity of PB leprosy cases by nested PCR with *folP*, *gyrA*, *rpoB*, *RLEP*, and *16SrRNA* and Enzyme-linked Immunospot Assay test (ELISPOT) with MMPII, NDO-BSA, and LID-1 antigens by detecting interferon gamma (IFN-γ) release. The overall positivity rates of genes tested in 300 clinical specimens were identified as 55% of *16SrRNA*, 59% of *RLEP*, 59.3% of *folP*, 57.3% of *rpoB*, 61% of *gyrA* while 90% of nested *folP*, 92.6% of nested *rpoB*, and 95% of nested *gyrA*, and 285 (95%) of at least one gene positive cases. For PB specimens, 95% PCR positivity was achieved by three tested genes in nested PCR. The data obtained from ELISPOT for three antigens were analyzed for IFN-γ expression with 600 subjects. Among 98 PB leprosy cases, the sensitivity of MMP II, LID-1, and NDO-BSA was 90%, 87%, and 83%, respectively, and the specificity was 90%, 91%, and 86%, respectively. The total number of cases positive for at least one antigen was 90 (91.8%) in PB, which is significantly higher than that in multibacillary (MB) leprosy (56.7%). The combination of multi-targets nested PCR and ELISPOT assay provides a specific tool to early clinical laboratory diagnosis of PB leprosy cases. The two assays are complementary to each other and beneficial for screening PB patients.

## Introduction

Leprosy is a chronic infectious disease caused by *Mycobacterium leprae*, which mainly affects the skin and peripheral nerves. Although multidrug therapy (MDT) has decreased the prevalence of leprosy worldwide, leprosy remains a public health problem with 200,000 new leprosy cases reported each year (Benjak et al., 2018). The diagnosis of leprosy usually depends on the clinical evaluation of leprosy-suspected patients. The disease is primarily diagnosed by a combination of clinical features, slit-skin smear, and histopathological examination (Scollard et al., 2006). In spite of these, these techniques are time consuming and have limited sensitivity, as clinical features and histopathology are often complex (Santos et al., 2013). As there are few skin lesions and pure neuritic type with the absence of bacilli in slit-skin smears, the diagnosis and discrimination of PB patients is challenging, particularly in the early stages. Therefore, it is necessary to implement a more sensitive, robust, and accurate diagnostic method to diagnose PB cases of leprosy.

Molecular assays, such as PCR, have proven to be powerful diagnostic tools in diagnosing pure neural leprosy and PB leprosy, where acid-fast bacilli are rare or even absent (Martinez et al., 2006; Banerjee et al., 2010). The PCR method has been applied to investigate possible leprosy sources for dissemination of *M. leprae* (Chokkakula et al., 2019; Chokkakula et al., 2020). With regard to diagnostics, nested PCR technology is considered to be at least 100 times more sensitive than PCR and microscopic detection. It is very important for the early diagnosis of patients with negative microscopy or differential diagnosis of lesions with inconclusive histopathology (Araujo et al., 2016). The diagnostic sensitivity of nested PCR assays is considered to be the highest for skin biopsies, with *M. leprae* DNA detection rates being reported from more than 80% of skin tissue samples of clinically MB cases and 30%–40% of BI negative PB cases. Among a range of possible gene targets, the *M. leprae*-specific element *RLEP*, *16srRNA*, *folP*, *gyrA*, and *rpoB* have been identified as the most suitable targets for diagnostic applications (Beissner et al., 2019). Each of these genes have been combined to develop for *M. leprae* detection and diagnostic assay to strengthen the diagnosis of critical cases of leprosy.

Interferon-gamma release assays (IGRAs), also known as the enzyme-linked immunospot (ELISPOT) assay, are based on the IFN-γ secretion by lymphocytes exposed to *M. tuberculosis*-specific antigens (Xu et al., 2015; Della Bella et al., 2018). Currently, very few studies have examined the utility of ELISPOT assays in *M. leprae*-infected individuals. Some of these studies explored the immune response in *M. leprae*-stimulated whole-blood assay (WBA) and examined the supernatant levels of IFN-γ stimulated with *M. leprae* from infected patients (Hungria et al., 2017; Chen et al., 2019b; Do Carmo Goncalves et al., 2020). Others focused on a panel of multiple *M. leprae* antigen-induced host markers by WBA (Chen et al., 2018). Recent studies reported that IFN-γ secretion induced by stimulation with *M. leprae* antigens achieved higher positive response rates in PB patients than in MB patients (Sampaio et al., 2011; Chen et al., 2018). These studies suggest that *M. leprae* antigen-specific IFN-γ secretion in the WBA has limited potential to discriminate PB patients; in fact, the detection of additional *M. leprae*-specific antigens and IFN-γ release by single cells should be investigated for their ability to diagnose and discriminate the PB patients.

This current study describes and evaluates application of a combined molecular and immunological diagnostic approach for the diagnosis of *M. leprae* infections, particularly for PB leprosy patients. We examined the significance of the nested PCR over conventional PCR in the diagnosis of leprosy cases and also conducted the ELISPOT assay to detect IFN-γ release to find out the importance of ELISPOT in the diagnosis of leprosy. The combined nested PCR and ELISPOT assays complement each other and are ancillary tests for the earlier detection and screening of *M. leprae*.

## Methods

### Ethics Statement

This study was approved by the institutional review and ethical committees of the Institute of Dermatology, Chinese Academy of Medical Sciences, China. Informed consent was obtained from all patients and healthy volunteers prior to blood and tissue collection.

### Patient Recruitment and Collection of Mycobacterial Specimens

A total of 300 clinical specimens of leprosy were obtained after clinical, slit-skin smear, and histopathological observations of clinically diagnosed new leprosy cases attended at the Institute of Dermatology during 2015–2020. The 300 specimens and their blood samples (202 MB and 98 PB biopsies) of clinically diagnosed leprosy cases and 150 household contact and 150 health donor blood samples were used for this study (**Supplementary Table S1**). Household contacts were recruited at the time of MDT initiation of the newly diagnosed leprosy cases. Diagnosis and prescription of PB and MB-multidrug therapy (MDT) regimen were performed as per WHO guidelines. The confirmation of the diagnosis is based on the patients’ medical history, clinical manifestations, slit-skin smear, and histological examinations; furthermore, each patient could be placed into a more rigid Ridley–Jopling (R–J) scale (LL, BL, BB, BT, and TT) (Kundu, 1979). Pure neuritic leprosy in patients causes insensitivity to fine touch, pain, and warmth, so we chose slit-skin smear (SSS) tissue sampling instead of punch biopsy for PCR-based diagnosis. After clinical examination, 6-mm punch biopsy samples and SSS samples were collected and fixed in 70% ethanol as per the WHO guidelines. The purified DNA from *M. leprae* of nude mice was used as a positive control and that from mycobacterial species containing *Mycobacterium tuberculosis*, *Mycobacterium fortuitum*, *Mycobacterium avium*, *Mycobacterium smegmatis*, *Mycobacterium intracellulare*, *Mycobacterium chimera*, and *Mycobacterium abscessus* as negative controls. Ten skin samples of *Pityriasis alba* causing common skin diseases were selected as negative controls. All these specimens were obtained from the Institute of Dermatology, Chinese Academy of Medical Sciences to evaluate the specificity of PCR assays.

### Genomic DNA Extraction

Ethanol-fixed biopsy and SSS samples from newly diagnosed leprosy and 10 P*. alba* samples were shifted to the core lab facility for processing, such as cutting of the biopsy into two halves, one washed twice with phosphate-buffered saline (PBS) followed by grinding by a glass Dounce homogenizer. According to the manufacturer’s instructions, genomic DNA isolation from tissue homogenate was performed by the DNeasy Blood and Tissue kit (Qiagen, Hilden, Germany, cat. no. 69504). The concentration and purity of the DNA samples were checked using the Nanodrop2000 (Thermo Fisher Scientific, Waltham, MA, USA), and these DNAs were used immediately or stored at −80°C.

### PCR Methods

Five genes, including *RLEP*, *folP*, *rpoB*, *gyrA*, and *16SrRNA* have been used in PCR-based diagnosis of leprosy (Chokkakula et al., 2019; Jiang et al., 2021). The primers for conventional PCR (first PCR) and nested PCR (second PCR) were designed by Premier5 Software and are summarized in **Supplementary Table S2**. The first PCR was performed with 50 μl of PCR reaction mixture containing the ingredients such as 25 μl of 1.25 U of Taq DNA polymerase mix (Promega, Norwalk, CT, USA), 2 μl of 5 pmol each of primers, 18 μl RNase-free water, and 5 μl genomic DNA (10 ng/μl). The PCR amplification conditions are follows: denaturation at 95°C for 5 min and 35 cycles of 95°C for 30 s, 65°C for 40 s, and 72°C for 1 min, followed by a final 10 min extension at 72°C. The Thai-53 strain *M. leprae* (Japan) was used as positive control. The nested PCR was performed in a total volume of 50 μl of PCR reaction mix containing 1 μl of PCR products from first PCR, 25 μl of 1.25 U of Taq DNA polymerase mix, 2 μl of 5 pmol each of primers, and 22 μl RNase-free water. The amplified products were electrophoresed on 2% agarose, and gel bands were observed by UV transillumination documentation system (BioRad, California, USA).

### DNA Sequencing

The PCR amplicons were purified and further sequenced at a local commercial facility (Tsingke Biological Technology Co., Ltd., Nanjing, China). Sequencing data were alimented to the National Center for Biotechnology Information (NCBI) nucleotide databases using basic local alignment sequence tool (BLAST) for the identification of pathogens.

### Isolation of PBMC by Ficoll–Paque Density Gradient Centrifugation

Peripheral blood mononuclear cells (PBMCs) were isolated from the peripheral blood using Ficoll–Paque PLUS (GE Healthcare, Washington, USA) as per the product instructions. Briefly, the blood was diluted with an equal volume of PBS. Twenty milliliters of diluted blood was layered over 10 ml of the Ficoll–Paque. The gradients were centrifuged at 540×*g* for 25 min at room temperature on a horizontal rotor with the minimum speed down. The PBMC interface was carefully removed by pipetting and washed with PBS by centrifugation at 300×*g* for 10 min. The PBMC pellets were incubated at room temperature for 10 min to lyse contaminating red blood cells, followed by a wash with PBS. Cell number and viability were determined using a Countess automated cell counter (Invitrogen). The PBMCs were cryopreserved in liquid nitrogen in fetal calf serum (FCS; Invitrogen) containing 10% dimethyl sulfoxide (DMSO; Thermo Fisher) and completed within 1 year by ELISPOT analysis to avoid loss of cellular activity.

### Antigen-Specific Antibody Detection by Enzyme-Linked Immunospot

Antigens, NDO-BSA, and LID-1 were obtained from the Infectious Disease Research Institute, Seattle, USA, and MMP-II was obtained from the Department of National Institute of Infectious Diseases, Japan. The 96-well plates were coated with IFN-γ captured antibody and incubated overnight at 4°C using human IFN-γ ELISPOT kits (BD, USA). Plates were then washed three times and blocked by culture medium with RPMI-1640 supplemented with 2 mm l-glutamine, 100 U/ml penicillin, 100 µg/ml streptomycin, and 10% FCS for 2 h at room temperature. For the cultured ELISPOT, the PBMCs were thawed, washed, and resuspended in culture medium. Cells were then used in the standard ELISPOT with 1×10^5^ cells/well, stimulated with the MMP-II, NDO-BSA, LID-1, and culture medium only or phytohemagglutinin (PHA; 5 μg/ml; Sigma-Aldrich) and cultured overnight at 37°C in a humidified 5% CO_2_ atmosphere. Plates were washed five times with PBS supplemented with 0.05% Tween-20 (PBST; Sigma-Aldrich). The biotinylated detection antibody for IFN-γ was added and incubated at room temperature for 1 h. After five washes with PBST, streptavidin–alkaline phosphatase conjugate was added, and the plates were incubated at room temperature for 1 h. The plates were then washed, added with 5-bromo-4-chloro-3-indolyl phosphate/nitro blue tetrazolium, and incubated at room temperature for 20 min. The wells were then washed several times under running water and air-dried. Spots were counted using an automated ELISPOT reader system (AID, Germany).

### Statistical Analysis

The ROC curve, 95% CI, and cutoff value were calculated using SPSS 14.0 Software. The mean number of spots from duplicate wells was adjusted to 1×10^5^ PBMC. The net spots per million PBMC were calculated as follows: mean number of spots per million PBMC in wells from antigens pool minus the mean number of spots per million PBMC in wells with culture medium only. For analysis of intra-assay cultured ELISPOT variability, the mean and SD were calculated for each individual set of duplicate wells, and each coefficient of variation (% CV) was determined. The inter-assay cultured ELISPOT variability was calculated as the % CV of the mean from duplicate wells from each assay performed on different days. Statistical analyses were performed using GraphPad Prism 5 (GraphPad Software Inc., CA, San Diego, USA). All tests were two-tailed, and p < 0.05 was considered statistically significant.

## Results

### Optimization of Nested PCR Conditions for *M. leprae*

To establish and optimize the nested PCR, *M. leprae* and other seven mycobacterial species were included in the study. These control samples were subjected to same processing steps as the tested specimens. All positive control samples showed positive results for the five tested genes, while none of the negative controls showed any positive results for these five tested genes in nested PCR (**Supplementary Table S3**). Both the first and second PCR assays found negative results for all seven mycobacterium control strains and 10 skin samples of *P. alba* (**Supplementary Figure S1**). The analytical specificity of these assays has been adequately demonstrated by these results.

### The Comparison of Conventional PCR With Nested PCR

**Figure 1:**
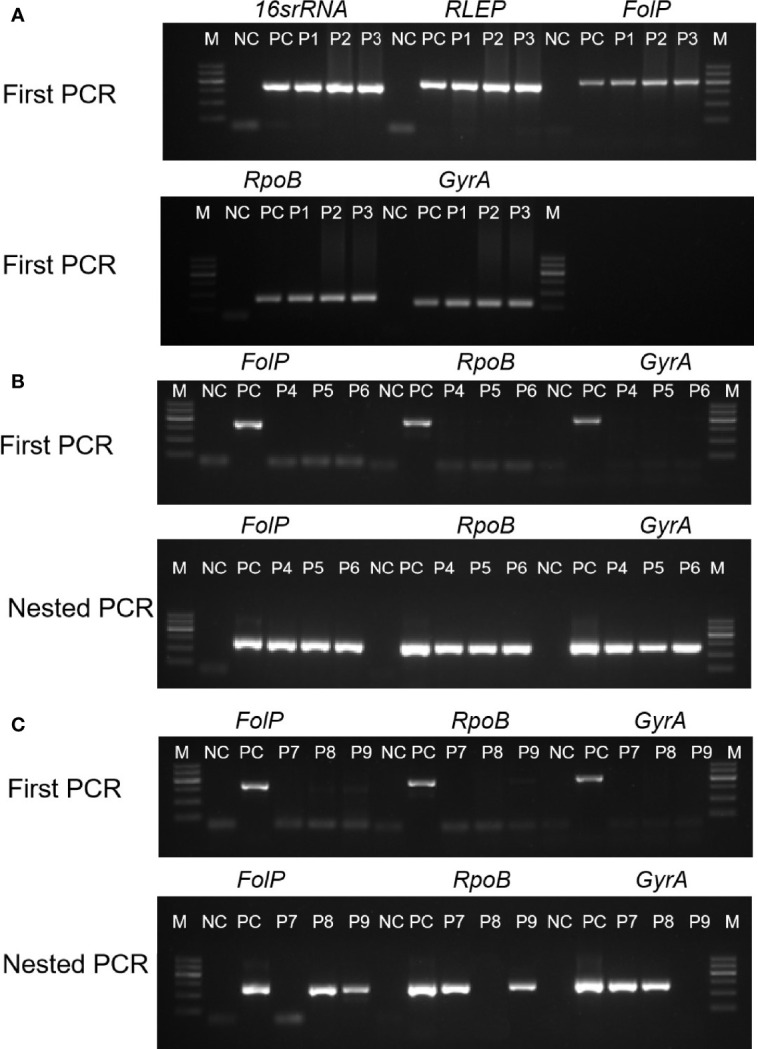
Examples of cases diagnosed *via* PCR and nested PCR were decisive in the clinically diagnosed new PB leprosy. The horizontal axis indicates the products of five DNA targets by agarose gel. Samples form clinically diagnosed PB leprosy could be detected by first PCR **(A)**, first PCR with nested PCR **(B)**, and nested PCR with one of target genes **(C)**. M, marker; NC, negative control; PC, positive control; P1–P9, clinical samples.

In the present study, we analyzed the clinical specimens obtained from 300 newly diagnosed leprosy patients, most of whom were 18–80 years of age. This study included 98 single skin lesion (PB) and pure neuritic leprosy cases. The 125 specimens (41.6%) were positive by acid-fast bacteria (AFB) staining, while 285 specimens (95%) showed positive results by targeting five genes of *M. leprae* by conventional and nested PCRs. Of the 300 patients, 125 cases (41.6%) have already been “confirmed” by AFB; conventional PCR assay confirmed the positivity of 183 cases (**Figure 1A** and **Supplementary Table S4**). In contrast, the nested PCR assay confirmed the positivity of 285 cases (95%) (**Supplementary Table S4**). Nested PCR significantly increased the detection rate than conventional PCR in the diagnosis of leprosy. All the positive results were identified as *M. leprae* by sequencing.

### The Positive Rate of PCR and Nested PCR of Five Target Genes of *M. leprae*

The overall positivity rates for the tested genes were 55% of *16SrRNA*, 59% of *RLEP*, 59.3% of *folP*, 57.3% of *rpoB*, and 61% of *gyrA*, while they were 90% of nested *folP*, 92.6% of nested *rpoB*, and 95% of nested *gyrA* (**Figures 1B, C** and **Table 1**). Therefore, the *gyrA* gene (95%) achieved the highest positive rate in nested PCR. The 194 cases showed positivity by nested PCR in MB patients specimens. The 12.2% (12 cases) positivity was detected by first PCR in single skin lesion and pure neuritic specimens, whereas 92.8% (91 cases) positivity was reported by nested PCR. The 285 cases with positive results were achieved by at least one of the tested genes in nested PCR. The PCR positivity of each specimen was validated by PCR amplification, sequencing, and blasting analysis.

**Table 1:** The overall positive rate of PCR and nested PCR using five different gene targets of *M. leprae* according to the type of skin specimens.

Clinical sample		Bacillary index	No. of samples	Positive *16SrRNA* (%)	Positive *RLEP* (%)	Positive *FolP* (%)	Positive *RpoB* (%)	Positive *GyrA* (%)	Positive Nested PCR *FolP* (%)	Positive Nested PCR *RpoB* (%)	Positive Nested PCR *GyrA* (%)
MB (>5 skin lesions)		2–5	202	162 (80)	170 (84)	166 (82)	162 (80)	166 (82)	190 (94)	194 (96)	194 (96)
PB	1–5 skin lesions	0–1	50	2 (4)	4 (8)	8 (15.4)	7 (14)	10 (19.2)	40 (80)	43 (82.7)	48 (92.3)
	Pure neuritic leprosy	0–1	48	1 (2)	3 (6.3)	4 (8.3)	3 (6.3)	7 (14.6)	40 (83.3)	41 (85.4)	43 (89.6)

### Diagnostic Performance of Three Proteins in ELISPOT

**Figure 2:**
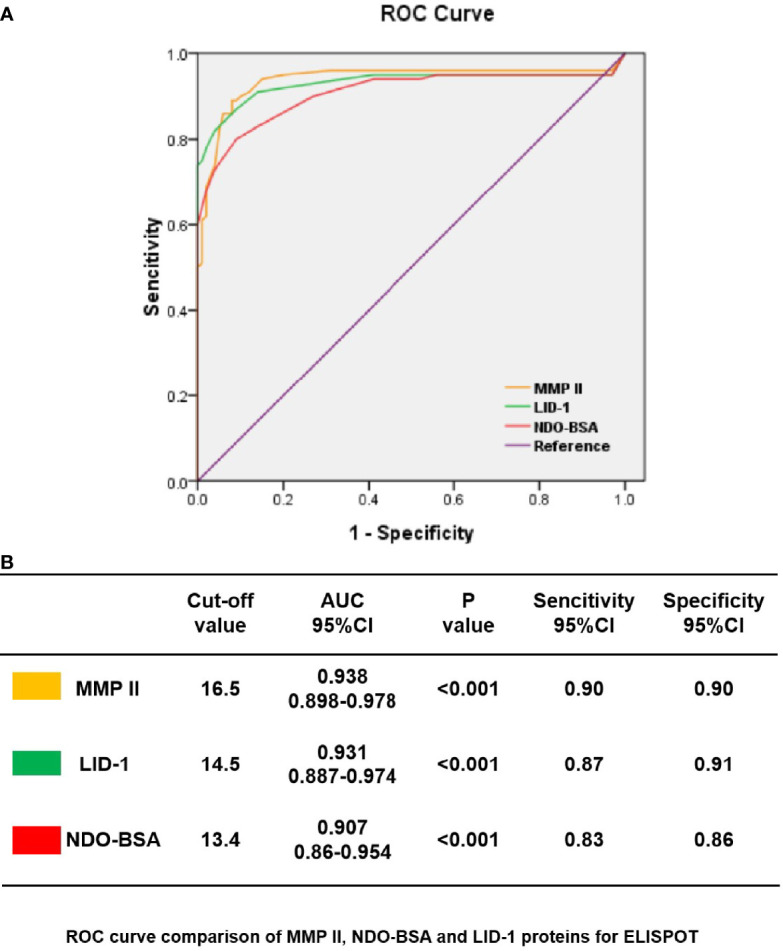
ROC curves showing the accuracy of host marker IFN-γ in discriminating among PB, MB, HHC, and HD. ROC curves for the accuracy of single markers IFN-γ induced by MMP II, NDO-BSA, and LID-1 to differentiate among PB patients and other controls **(A)**. ROC curves for markers that differentiated PB patients with health controls with p<0.001 are shown; the cutoff values with MMP II, NDO-BSA and LID-1 were 16.5, 13.4, and 14.5 respectively **(B)**.

**Figure 3:**
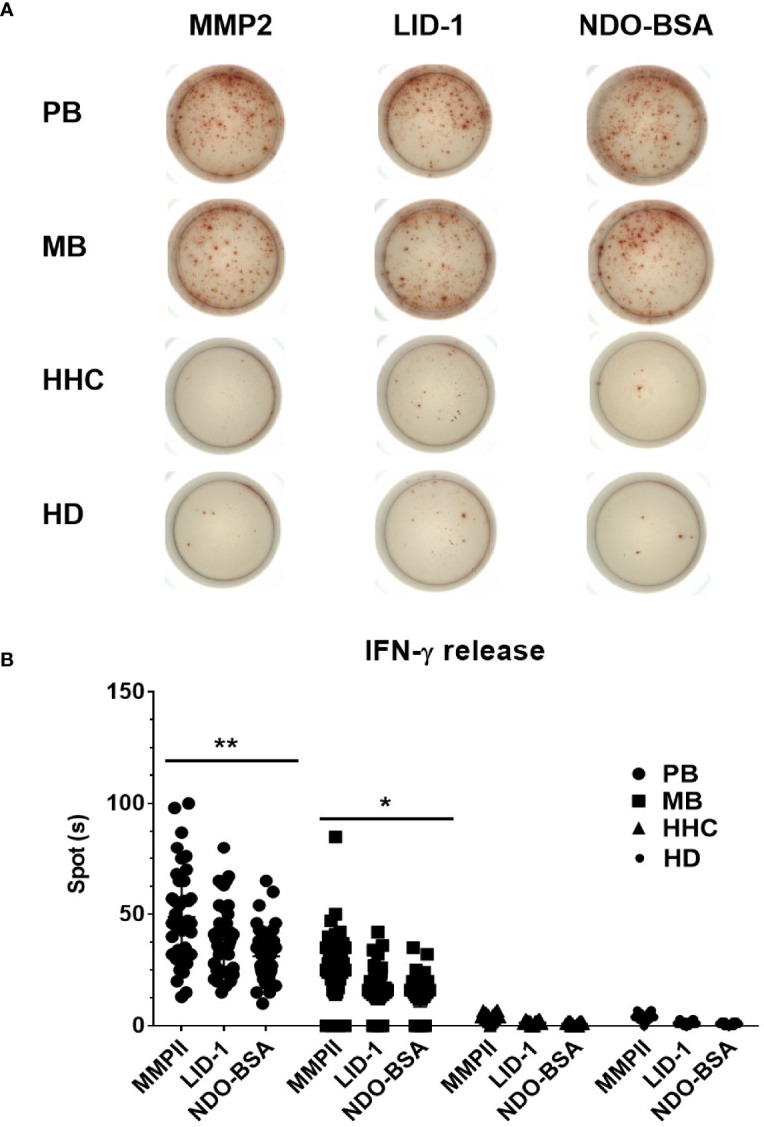
Comparative analysis of IFN-γ expression of ELISPOT in blood samples. Clinically diagnosed PB leprosy (n = 98), clinically diagnosed MB leprosy (n = 202), HHC (n = 150), and HD (n = 150) were detected with IFN-γ expression using ELISPOT **(A)** and analyzed by GraphPad Prism **(B)**. *p<0.05, **p<0.01.

First, we choose 50 MB patients, 50 PB patients, and 50 household contact from MB and PB patients, and 50 health donors by ELISPOT for each antigen was analyzed through ROC curve (**Figure 2A**). The area under curve (AUC) is an indication of the diagnostic sensitivity of the antigen variant. The AUC was 0.938 (0.898–0.978) for MMP II protein as compared to 0.931 (0.887–0.974) in the case of LID-1 protein, 0.907 (0.86–0.954) for NDO-BSA. The cutoff value of MMP II, LID-1, and NDO-BSA is 16.5, 14.5, and 13.4, respectively (**Figure 2B**). Using MMP II, as antigen to perform ELISPOT, the sensitivity is 90%, and the specificity is 90%. The sensitivity of LID-1 is 87%, while the specificity increased to 91%. When using NDO-BSA as antigen to perform ELISPOT, the sensitivity is 83%, and the specificity is 86% (**Figure 2B**). All 600 subjects included in the analysis with three antigens stimuli produced measurable IFN-γ responses in ELISPOT assays. Of 98 PB patients, 90 (91.8%) were ELISPOT positive, and the follow-up of the 202 MB patients showed 56.7% positivity for ELISPOT (**Table 2**). The 150 household contacts and 150 health donors reported 3.3% positivity (**Table 2**). The magnitude of responses to three stimuli was significantly higher IFN-γ responses among PB patients compared to MB patients and controls (**Figures 3A, B** **)**. Total number of positive specimens for at least one of the three antigens were 90 (91.8%) in PB cases and 115 (56.7%) in MB cases. The diagnostic rate of PB was significantly higher than MB by ELISPOT assay. The detection rate of three proteins in ELISPOT assay revealed that the humoral immunity induced by the proteins is little affected by the polymorphism of the three proteins.

**Table 2:** The overall positive rate of ELISPOT using three antigens of *M. leprae* in PBMC.

Clinical sample		Bacillary index	No. of samples	Positive MMP II (%)	Positive NDO-BSA (%)	Positive LID-1 (%)	p-value
	MB	2–5	202	110 (54.7)	115 (56.7)	113 (56)	0.880638
PB	1–5 skin lesions	0–1	50	46 (92)	45 (90)	44 (88)	0.800737
	Pure neuritic leprosy	0–1	48	44 (91.7)	43 (89.6)	43 (89.6)	0.923928
Household contact		–	150	5 (3.3)	4 (2.7)	4 (2.7)	0.923845
Health donor		–	150	5 (3.3)	3 (2)	4 (2.7)	0.773485

### Comparison of Nested PCR and ELISPOT Assays Results in PB Leprosy Cases

The five genes and three stimuli were analyzed by PCR and ELISPOT assay in 300 specimens. Of the 202 MB cases, 194 (94%) were positive on nested PCR, and 115 (56.7%) cases were positive on ELISPOT. Among the 202 MB cases, 79 (39.1%) were negative on ELISPOT but positive on nested PCR (**Table 3**). On follow-up of 98 PB cases, 90 (91.8%) cases were identified as positive on ELISPOT, while 91 (92.9%) cases reported as positive on nested PCR. In addition, six samples were positive on nested PCR and negative on ELISPOT, and three cases were positive on ELISPOT and negative on nested PCR (**Table 3**). Among the 300 household contacts and healthy donors, 10 cases were detected above the cutoff values for ELISPOT. For conventional PCR comparison with nested PCR in PB cases, the positive detection rate of nested PCR was significantly improved (p<0.01) (**Table 4**). Both nested PCR and ELISPOT assay have their own significance, and the combination of these two assays could have complemented each other in diagnosing the PB leprosy cases.

**Table 3:** Diagnostic performance of nested PCR and ELISPOT assay of 300 leprosy cases by diagnostic category.

All patients (n = 300)		Nested PCR positive	ELISPOT positive	Nested PCR positive/ELISPOT negative	ELISPOT positive/nested PCR negative
MB		194/202 (94%)	115/202 (56.9%)	79 (37.6%)	0
	1–5 skin lesions	48/50 (92.4%)	46/50 (92%)	4 (8%)	2 (4%)
PB	Pure neuritic leprosy	43/48 (89.6%)	44/48 (91.7%)	2 (4.2%)	1 (2.1%)
	Not leprosy	0/10 (0%)	–	–	–
Household contact		–	5/150 (3.3%)	–	–
Health donor		–	5/150 (3.3%)	–	–

**Table 4:** Comparison of the results of molecular and ELISPOT assays using clinical samples of clinically diagnosed new PB leprosy patients and healthy donor.

Method	Gene or antigen (loci)		Positive value		p-value
PCR vs. nested PCR	PCR	Nested PCR	PCR	Nested PCR	
	*FolP* (Outer primers)	*FolP* (Inner primers)	12 (12.2%)	80 (81.6%)	0.0001
	*RpoB* (Outer primers)	*RpoB* (Inner primers)	10 (10.2%)	84 (85.7%)	0.0001
	GyrA (Outer primers)	*GyrA* (Inner primers)	17 (17.3%)	91 (92.9%)	0.0001
	At least one gene is positive	At least one gene is positive	17 (17.3%)	91 (92.9%)	0.0001
PCR vs. ELISPOT	PCR	ELISPOT	PCR	ELISPOT	
	At least one gene is positive	At least one antigen is positive	17 (17.3%)	90 (91.8%)	0.0001
Nested PCR vs. ELISPOT	nested PCR	ELISPOT	nested PCR	ELISPOT	
	At least one gene is positive	At least one antigen is positive	91 (92.9%)	90 (91.8%)	1.0000

## Discussion

The diagnosis of PB leprosy is always a challenge mainly in pure neuritic and few skin lesions where there is absence of bacilli in slit-skin smear, which usually leads to misdiagnosis due to lack of adequate laboratory diagnostic technique. For these reasons, it is needed that a specimen can be simultaneously subjected to various diagnostic tests, such as histopathological and microbiological examinations. Here, we reported a higher detection rate of PB leprosy in clinical specimens of clinically diagnosed new leprosy cases using nested PCR and ELISPOT assays. The key findings are as follows. First, positivity rates of 90% of *folP*, 92.6% of *rpoB*, and 95% of *gyrA* were achieved by nested PCR, which were significantly higher than that of conventional PCR in clinically diagnosed new PB leprosy. The highest positive rate in nested PCR was achieved by *gyrA* gene. Second, the positivity rates for PB leprosy specimens of MMP II, LID-1, and NDO-BSA were 90%, 91%, and 86%, respectively, by ELISPOT assay. Third, for 98 PB cases, 90 (91.8%) cases were identified as positive on ELISPOT, while 91 (92.9%) cases were reported as positive on nested PCR. Fourth, six samples were positive on nested PCR and negative on ELISPOT, and three cases were positive on ELISPOT and negative on nested PCR in clinically diagnosed new PB leprosy. Through the observation of disease treatment, we found that the positive specimens of disease diagnosis were effectively treated.

We investigated the performance of diagnostic accuracy and compared PB with MB cases. PB cases were evaluated in this study by multilocus combination of five tested genes *gyrA*, *folP*, *rpoB*, *16srRNA*, and *RLEP*, which indicated increasing detection rate by 60% on nested PCR. The use of nested PCR increased the sensitivity of a PB diagnosis and had higher specificity and accuracy than conventional PCR. Nested PCR method was involved in the testing of *M. leprae* drug resistance and other *Mycobacterium* species or non-mycobacterial species (Chen et al., 2019a; Wu et al., 2008; Sinha et al., 2016), however, without further analysis for the detection of PB leprosy. We optimized nested PCR through primer design and facilitated an early diagnosis of PB cases, which were often difficult to diagnose with the available standard methods. Since both false-negative and false-positive results have also been reported for nested PCR and there is a possibility of cross-contamination, care should be taken throughout the DNA extraction procedure and PCR (Araujo et al., 2016). The effective dilute product of the first reaction of PCR was used in nested PCR, which effectively increased the sensitivity and reduced the concentration of the remaining inhibitory substances (Kim et al., 2011; Janardhanan et al., 2014). Maintaining the environment for molecular amplification and using several targets in nested PCR could improve the yield and eliminate the false-positive or false-negative results, which were more common in single target gene PCR performances. To eliminate false results, we used the control PCR reaction system for confirmed leprosy and obtained positive nested PCR results for all leprosy-confirmed cases, with multiple loci supporting each other. We tried to get more positive results by using five various target genes for suspected leprosy specimens, respectively. Among these nested PCR-targeting genes, the *gyrA* showed more sensitivity in comparison to other tested genes (Araujo et al., 2014). The results showed that multilocus nested PCR could effectively improve the diagnostic rate of PB leprosy.

ELISPOT has been reported as an important alternative immunological tool in the diagnosis of tuberculosis (Yen et al., 2013; Deng et al., 2021). Some pathogenic protein antigens were used in the serological diagnosis of leprosy, such as *M. leprae* antigens MMP II, NDO-BSA, and LID-1 (Wang et al., 2015; Le et al., 2018). We induced host immune IFN-γ responses by antigenic stimulation in PBMC for the diagnosis of PB patients and discrimination between MB patients, household contacts, or health donors. The IFN-γ was activated significantly higher in PB leprosy than in MB with the stimulation of three proteins by ELISPOT assay. The detection rate of MMP II was slightly higher, but there was no difference among three proteins used in ELISPOT assay, revealing that the humoral immunity induced by these proteins were little affected by the polymorphism of the three proteins. The findings presented here applied to both PB and MB patients; however, PB patients showed a higher positive rate using three antigens of *M. leprae* in PBMC in the ELISPOT assay than patients with MB. The reason for this difference may be influenced by immunosuppression in the host (Chen et al., 2019b; Freitas et al., 2015). The MB patients are often immunocompromised, and immunosuppression itself may contribute to the low sensitivity of the ELISPOT, although the immune status of these MB patients we have enrolled is not completely clear. This is in line with previous published studies showing that PB patients produce IFN-γ, and MB patients exhibit a weak/absent response. Cytokines and chemokines, such as IFN-γ, may fluctuate during immune responses to *M. leprae* antigens (Freitas et al., 2015). Moreover, some studies have shown that patients who are immunocompromised are more sensitive to ELISPOT assay than immunocompromised tuberculosis patients (Lee et al., 2013). Therefore, further studies are needed to evaluate the diagnostic performances of ELISPOT assay in immunocompromised patients. The detection rate of ELISPOT was similar to that of nested PCR in PB cases and could complement each other in the diagnosis of leprosy cases.

Collectively, we suggest that this combined nested PCR and ELISPOT assay be more beneficial to PB patients with atypical clinical manifestations and are difficult to diagnose. For MB patients, the accuracy and specificity of nested PCR are enough for diagnosis. There are several limitations to our study. As the number of certain specimen types are limited by resource constraints and clinical specimen collection, the confidence intervals of our test results have been narrower if we had employed a larger cohort and more specimen types. Nevertheless, this is the largest PB diagnostic study utilizing nested PCR and ELISPOT assays ever undertaken. Since there is a long period from infection to onset of leprosy, we have not been able to do more prospective studies to clearly detect new leprosy infection (such as close contacts of these leprosy patients) or predict progression to active disease, and the ability to diagnose latent infections. More specimens are needed in future studies to investigate the efficacy of target genes and antigens in the diagnosis.

In conclusion, this is the first study to evaluate the clinical diagnostic value of nested PCR in combination with the ELISPOT in immunological and molecular microbiological approach for the diagnosis of PB leprosy with pure neuritic and few skin lesions. The combined nested PCR and ELISPOT assays complement each other and are ancillary tests for the earlier detection and screening of *M. leprae*.

## Data Availability Statement

The raw data supporting the conclusions of this article will be made available by the authors, without undue reservation.

## Ethics Statement

The studies involving human participants were reviewed and approved by the institutional review and ethical committees of the Institute of Dermatology, Chinese Academy of Medical Sciences, China. The patients/participants provided their written informed consent to participate in this study. Written informed consent was obtained from the individual(s) for the publication of any potentially identifiable images or data included in this article.

## Author Contributions

All authors listed have made a substantial, direct, and intellectual contribution to the work and approved it for publication.

## Funding

This work has been supported by the Jiangsu Provincial Science and Technology Project (BE2018619), National Natural Science Foundation of China (grant 81371751, 81972950), Chinese Academy of Medical Sciences Innovation Fund for Medical Science (2016-I2M-1-005, 2017-I2M-B&R-14), and Zhejiang Provincial Natural Science Foundation of China under Grant No. LGF19H260001.

## Conflict of Interest

The authors declare that the research was conducted in the absence of any commercial or financial relationships that could be construed as a potential conflict of interest.

## Publisher’s Note

All claims expressed in this article are solely those of the authors and do not necessarily represent those of their affiliated organizations, or those of the publisher, the editors and the reviewers. Any product that may be evaluated in this article, or claim that may be made by its manufacturer, is not guaranteed or endorsed by the publisher.
