# Correction: Utility of multi-target nested PCR and ELISPOT assays for the detection of paucibacillary leprosy: a possible conclusion of clinical laboratory misdiagnosis

**DOI:** 10.3389/fcimb.2025.1701705

**Published:** 2026-01-30

**Authors:** Haiqin Jiang, Ying Shi, Santosh Chokkakula, Wenyue Zhang, Siyu Long, Zhenzhen Wang, Wenming Kong, Heng Long, Limei Wu, Lihua Hu, Qiang Yao, Hongsheng Wang

**Affiliations:** 1 Department of Mycobacterium, Jiangsu Key Laboratory of Molecular Biology for Skin Diseases and STIs, Institute of Dermatology, Chinese Academy of Medical Sciences & Peking Union Medical College, Nanjing, China; 2 National Centre for STD and Leprosy Control, China CDC, Nanjing, China; 3 Centre for Global Health, School of Public Health, Nanjing Medical University, Nanjing, China; 4 Department of Microbiology, Chungbuk National University College of Medicine, and Medical Research Institute, Cheongju, Republic of Korea; 5 Department of Leprosy Control, Zhejiang, Provincial Institute of Dermatology, Zhejiang, China; 6 Department of Leprosy Control, Wenshan institute of Dermatology, Wenshan, China

**Keywords:** diagnosis, PCR, nested PCR, ELISPOT, paucibacillary leprosy

There was a mistake in **Figure 3A** as published. Four blot patches from the PB, MB and HD groups were mistakenly duplicated. The corrected **Figure 3A** using the original, correct data for the groups appears below.

**Figure 3:**
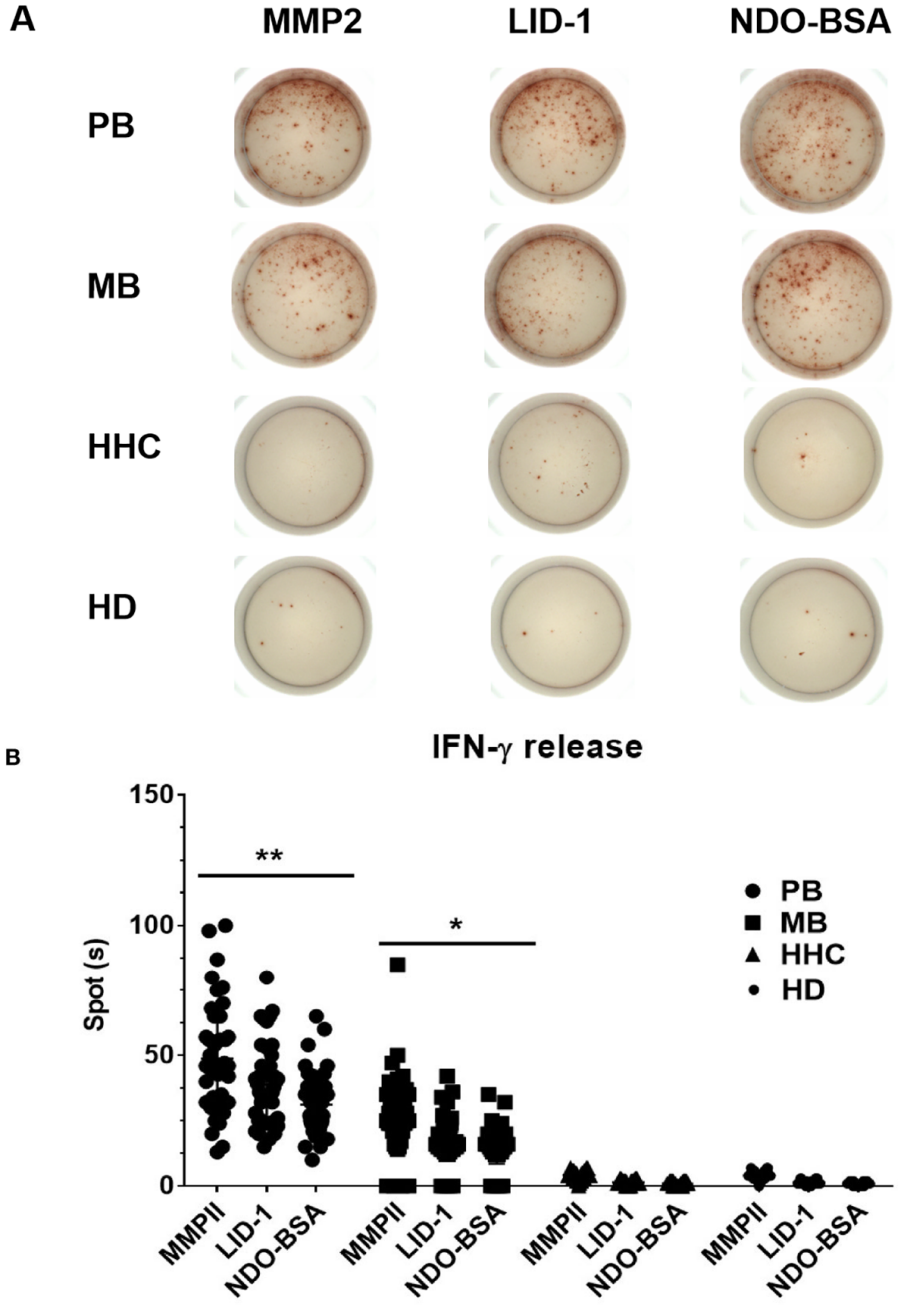
Comparative analysis of IFN-γ expression of ELISPOT in blood samples. Clinically diagnosed PB leprosy (n = 98), clinically diagnosed MB leprosy (n = 202), HHC (n = 150), and HD (n = 150) were detected with IFN-γ expression using ELISPOT **(A)** and analyzed by GraphPad Prism **(B)**. *p<0.05, **p<0.01.

The original version of this article has been updated.

