# Supplementary material for: Utility of Multi-target Nested PCR and ELISPOT Assays for the Detection of Paucibacillary Leprosy: A Possible Conclusion of Clinical Laboratory Misdiagnosis
Source: Front Cell Infect Microbiol. 2022 Apr 11;12:814413. doi: 10.3389/fcimb.2022.814413 (PMC9036522; doi:10.3389/fcimb.2022.814413)
Supplement: Supplementary file 1 [file Table_1.doc]

**Supplementary Tables**

**Supplementary Table 1. A demographic details of enrolled leprosy patients and control group**

| **Category** | | **Gender**  **(Male/Female)** | **Age** | **Case type** | **Bacillary index** | **Total number of demographics** | **P value** |
| --- | --- | --- | --- | --- | --- | --- | --- |
| MB | | 100 | 25-66 | New | 2-5 | 202 | 0.9208 |
|  |  | 102 | 20-65 |  |  |  |  |
| PB | 1-5 skin lesions | 24 | 25-66 | New | 0-1 | 50 | 0.8416 |
|  |  | 26 | 27-60 |  |  |  |  |
|  | Pure neuritic leprosy | 25 | 29-70 | New | 0-1 | 48 | 0.8384 |
|  |  | 23 | 26-75 |  |  |  |  |
| Household contact | | 74 | 20-60 | - | - | 150 | 0.9081 |
|  |  | 76 | 20-65 |  |  |  |  |
| Healthy donor | | 78 | 19-59 | - | - | 150 | 0.5638 |
|  |  | 72 | 20-62 |  |  |  |  |
| Pityriasis alba | | 6 | 30-55 | New | - | 10 | 0.7324 |
|  |  | 4 | 28-49 |  |  |  |  |

**Supplementary Table 2. Primer sequences for five target genes**

| **Gene** | **Primer sequence** | | **Amplicon size (bp)** | **Annealing temperature** |
| --- | --- | --- | --- | --- |
| *16SrRNA* | Primers | Forward: 5′-CGGAAAGGTCTCTAAAAAATCTT-3′ | 171 | 58 |
|  |  | Reverse: 5′-CATCCTGCACCGCAAAAAGCTT-3′ |  |  |
| *RLEP* | Primers | Forward: 5′-TGCATGTCATGGCCTTGAGG-3′ | 129 | 59 |
|  |  | Reverse: 5′-CACCGATACCAGCGGCAGAA-3′ |  |  |
| *FolP* | Outer primers | Forward: 5′-CAATTCGTTCTCAGATGGCGG-3′ | 343 | 58 |
|  |  | Reverse: 5′-CATCAACACCCACGCAACAC-3′ |  |  |
|  | Inner primers | Forward: 5′-CTTGATCCTGACGATGCTGT-3′ | 254 | 58 |
|  |  | Reverse: 5′-CCACCAGACACATCGTTGAC-3′ |  |  |
| *RpoB* | Outer primers | Forward: 5′-AGCGGATGACCACCCAGGA-3′ | 406 | 58 |
|  |  | Reverse: 5′-TCTTCCTCGTCAGCGGTCAA-3′ |  |  |
|  | Inner primers | Forward: 5′-GTCGAGGCGATCACGCCGCA-3′ | 279 | 58 |
|  |  | Reverse: 5′-CGACAATGAACCGATCAGAC-3′ |  |  |
| *GyrA* | Outer primers | Forward: 5′-GCGCAGCTATATTGATTACGCG-3′ | 387 | 58 |
|  |  | Reverse: 5′-GCTCCAGTAACGATATCACC-3′ |  |  |
|  | Inner primers | Forward: 5′-AAGTCCGCGATGGTCTCAAA-3′ | 263 | 58 |

**Supplementary Table 3. Comparison of PCR targeting five genes among *M. leprae* with other mycobacteria**

| **Specimens** | ***16SrRNA*** | ***RLEP*** | ***FolP*** | ***RpoB*** | **GyrA** | **Nested PCR *FolP* (254 bp)** | **Nested PCR *RpoB* (279 bp)** | **Nested PCR *GyrA* (263 bp)** |
| --- | --- | --- | --- | --- | --- | --- | --- | --- |
| *M.leprae* | + | + | + | + | + | + | + | + |
| *M. tuberculosis* | - | - | - | - | - | - | - | - |
| *M. fortuitum* | - | - | - | - | - | - | - | - |
| *M. avium* | - | - | - | - | - | - | - | - |
| *M. intracellulare* | - | - | - | - | - | - | - | - |
| *M. abscessus* | - | - | - | - | - | - | - | - |
| *M. smegmatis* | - | - | - | - | - | - | - | - |
| *M. chimera* | - | - | - | - | - | - | - | - |

**Supplementary Table 4. Comparison of PCR and nested PCR results among *M.leprae* specimens with different gene targets.**

| **No. of specimens** | ***16SrRNA*** | ***RLEP*** | ***FolP*** | ***RpoB*** | ***GyrA*** | **Nested PCR *FolP* (254 bp)** | **Nested PCR *RpoB* (279 bp)** | **Nested PCR *GyrA* (263 bp)** |
| --- | --- | --- | --- | --- | --- | --- | --- | --- |
| 165 | + | + | + | + | + | + | + | + |
| 7 | - | + | + | + | + | + | + | + |
| 5 | - | + | + | - | + | + | + | + |
| 1 | - | - | + | - | + | + | + | + |
| 5 | - | - | - | - | + | + | + | + |
| 87 | - | - | - | - | - | + | + | + |
| 8 | - | - | - | - | - | - | + | + |
| 7 | - | - | - | - | - | - | - | + |
| 15 | - | - | - | - | - | - | - | - |
